# Origami-based cellular metamaterial with auxetic, bistable, and self-locking properties

**DOI:** 10.1038/srep46046

**Published:** 2017-04-07

**Authors:** Soroush Kamrava, Davood Mousanezhad, Hamid Ebrahimi, Ranajay Ghosh, Ashkan Vaziri

**Affiliations:** 1 Department of Mechanical and Industrial Engineering, Northeastern University, Boston, MA 02115, USA; 2 Department of Mechanical and Aerospace Engineering, University of Central Florida, Orlando, FL 32816, USA

## Abstract

We present a novel cellular metamaterial constructed from Origami building blocks based on Miura-ori fold. The proposed cellular metamaterial exhibits unusual properties some of which stemming from the inherent properties of its Origami building blocks, and others manifesting due to its unique geometrical construction and architecture. These properties include foldability with two fully-folded configurations, auxeticity (i.e., negative Poisson’s ratio), bistability, and self-locking of Origami building blocks to construct load-bearing cellular metamaterials. The kinematics and force response of the cellular metamaterial during folding were studied to investigate the underlying mechanisms resulting in its unique properties using analytical modeling and experiments.

Origami, the ancient Japanese art of paper folding, relies on seemingly straightforward operations of concerted folding of a flat sheet of paper to produce incredibly complicated geometrical objects. This relatively simple control of topology makes Origami an important conceptual paradigm for deployable structures across a wide spectrum of applications. This includes several recent demonstrations in areas as diverse as deployable solar panels^1^,^2^, fold-core sandwich panels^3^,^4^, three-dimensional (3D) cell-laden microstructures^5^, flexible medical stents^6^, flexible electronics^7^, soft pneumatic actuators^8^, and self-folding robots and structures^9^,^10^,^11^. Furthermore, periodic cellular metamaterials have been recently designed by assembling foldable Origami units (i.e., sheets or tubes) which tessellate to fill the 3D space^12^,^13^,^14^,^15^,^16^,^17^. In addition, Origami has found applications in designing mechanical metamaterials with tunable stiffness, auxeticity, bistability, load bearing capacity and self-folding features^14^,^15^,^18^,^19^,^20^,^21^,^22^.

Although an Origami construction relies on a mechanically simple folding operation, discovering the exact sequence of folds for a desired behavior is a combinatorically intractable problem^23^,^24^,^25^. In this context, simplification is possible through an intricate coupling of topology and mechanical compatibility to design periodic fold sequence that can be repeated to create such Origami^26^,^27^. An example is the pioneering work of Tachi and Miura^13^, who introduced a type of rigid Origami based on the previously-proposed Miura-ori fold^28^. Miura-ori is a single degree of freedom (DOF) rigid-foldable Origami shown in Fig. 1(a) – left image. The four crease lines of Miura-ori which result in one mountain and three valley folds define four identical parallelograms with adjacent sides defining an acute angle, *α* [shown in Fig. 1(a) – left image]. As the flat sheet deforms, these parallelograms become inclined to each other which can be quantified in terms of dihedral angles, 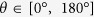, 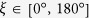, or the angle between the mountain and front valley folding lines, 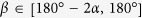. Due to the geometrical constraints, only one of these angles (*θ, ξ*, or *β*) is independent and can then be used to represent the single DOF of the system in analysis. For example, *β* and *ξ* can be expressed in terms of *θ*, and the constant angle, *α*, using the following relationships [see Supporting Information for details]:


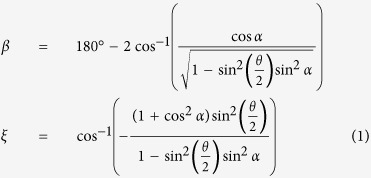


Putting Miura-ori units next to each other results in a Miura-ori sheet construction while retaining its single DOF properties and rigid-foldability. Stacking and bonding Miura-ori sheets along fold lines are shown to form cellular metamaterials with a single DOF that can be machined into any desired shape while preserving its folding motion^14^,^29^.

In this work, we propose a new class of Origami-based cellular metamaterials with a wide range of interesting properties such as auxeticity, bistability, foldability, and self-locking. We start our design with putting together four Miura-ori folds as shown in Fig. 1(a) – middle image. First, two Miura-ori units were positioned in a zigzag pattern, then mirrored to form a symmetric structure, preserving the single DOF, inherent to the original Miura-ori fold. Based on this design, we fold a single sheet of paper to construct a ‘first-order element’ that will be used in developing the Origami-based cellular metamaterial, Fig. 1(a) – right image. It is noteworthy that folding of the first order element, for example by changing *θ*, results in change in its overall length; however, the left and right parts of the element stay aligned, independent of the folding level.

First-order elements can be attached together in three different ways, shown in Fig. 1(b), to make a ‘second-order element’. From these three configurations, only the configuration shown on the right can be made by folding a single sheet of paper, and the other two configurations can be constructed by attaching the two first-order elements. The angle between the two segments in each second-order element is denoted by *γ*_1_, *γ*_2_, and *γ*_3_, which can be calculated as 180° − *β*, 180° − *β*, and *β*, respectively (recall from Fig. 1(a) that *β* is an angle varying between 180° − 2*α* and 180°). Considering *γ*_1_, *γ*_2_, and *γ*_3_ as internal angles, these second-order elements can be connected to generate contiguous geometrically closed-loop elements with many different topologies with the following geometrical constraints: 1. Second-order elements with *γ*_1_ and *γ*_2_ cannot be adjacent, 2. The two sides of the second-order element with *γ*_3_ cannot be connected to two identical elements with *γ*_1_ or *γ*_2_. Note that ignoring these geometrical constraints will result in closed-loop elements with at least one external angle with γ_1_ or γ_2_ or γ_3_ value (i.e., closed-loop elements with at least one internal angle not equal to γ_1_ or γ_2_ or γ_3_). Figure 1(c) shows three possible quadrangular configurations that satisfy above constraints.

We now prove that from all possible close-loop elements only one arrangement leads to a rigid-foldable geometry. For each closed-loop element with *n* sides, the summation of all internal angles must be equal to 180° × (*n* − 2), where *n* is the number of first-order elements used to construct the closed-loop element. Denoting *m*_*i*_ (*i* = 1, 2, 3) as the number of *γ*_*i*_ (*i* = 1, 2, 3) angles (i.e., *n* = *m*_1_ + *m*_2_ + *m*_3_) yields the following geometrical relationship:


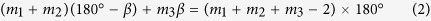


To achieve a foldable configuration, the left hand side of Equation (2) must be independent of the folding variable, *β* (note that the right hand side of the equation is a constant and independent of *β*). This yields *m*_1_ + *m*_2_ = 2 and *m*_3_ = 2, meaning that the only possible foldable configuration is a ‘quadrangle’ (*n* = 4). The examples provided in Fig. 1(c) are the only configurations that satisfy the Equation (2). The left and middle configurations can only built for *β* = 90°, while the right configuration can be built for any value of 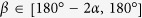. This means that the left and middle configurations are rigid and the only possible foldable polygon is the jigsaw-puzzle-like unit cell highlighted in green (see Supporting Information for further discussions on the rigidity of unit cells). All other possible configurations of triangular, quadrilateral, and hexagonal closed-loop elements (i.e., the only 2D shapes which can individually tessellate the 2D space to form periodic geometries), formed by different types of second-order elements introduced in Fig. 1(b), are given in Fig. 2. Note that all these elements are rigid (i.e., non-foldable), since they don’t satisfy Equation (2), however, they can be used as building blocks to construct rigid tessellations such as the well-known ‘Kagome’ structure made from triangular and hexagonal elements (see Supplementary Fig. S2 for an illustration of the structure).

It is essential to employ a connecting mechanism to link the adjacent unit cells of a lattice structure together, to form the final configuration of the system. An example of this mechanism is using an adhesive material to connect the unit cells together, however, this may affect the foldability of the structure by restricting degrees of freedom of the system, which will definitely alter the geometrical and mechanical properties of the final assembly. Here, we introduce an embedded self-locking mechanism into the proposed foldable unit, bonding the adjacent units together, which originates from the locking of first-order elements as shown in Fig. 3(a). To ensure fitting of one first-order element into another, each element must have a folding level corresponding to *β* > 90°. Once a contact is established between the two elements, self-locking can manifest by decreasing the folding angle to *β* < 90°, as for example is achieved in Fig. 3(a) – right image, by applying an out-of-plane compression.

The foldable closed-loop element (i.e., Fig. 1(c) – right image) can be stacked in the out-of-plane direction to create a foldable tubular topology, which then can be used as building blocks to construct a cellular metamaterial, Fig. 3(b). The self-locking feature of the first-order elements described above gets transferred to these building blocks and similarly gets activated for folding levels with *β* < 90°. Note that this locked state would impose effective contact strength between the building blocks in addition to simple frictional assembly. To this end we subjected a prototype, made of paper, to tension, when in locked and unlocked states, Fig. 3(c) (see Supporting Information for details on the experiments). When in the unlocked state, the structure exhibits no force resistance [i.e., force ~ 0 (N)], while in the locked state the structure shows noticeable resisting force [i.e., force ~ 35 (N)] before locking fails (see Supporting Information and Movie). Note that the resisting force strongly depends on folding level as well as the mechanical properties (i.e., elasticity) of the parent material which the plates are made of. However, the main goal of these experiments was to demonstrate the effect of the embedded self-locking mechanism on the structural resistance against the applied in-plane tensile load by comparing their resisting force in unlocked versus locked configurations. In theory, since the plates are assumed to be rigid, the resisting force will be infinite in the locked configuration.

The behavior and properties of the cellular metamaterial, which exhibits periodicity in both in-plane as well as out-of-plane directions can be analytically evaluated by assuming an infinite repetition of a representative volume element (i.e., RVE; same as the closed-loop element) of the cellular metamaterial, Fig. 4(a) – left and middle images. Thus, we investigate the kinematics and kinetics of the cellular metamaterial by analyzing the closed-loop element during folding. Figure 4(a) shows top and side views of the closed-loop element as well as the geometrical characteristics of the constituting first-order element introduced earlier. The in-plane diagonals, *D*_1_ and *D*_2_, and out-of-plane height, *H*, of the closed-loop element at an arbitrary level of folding, illustrated in Fig. 4(a), are given in terms of the geometry of the underlying Miura-ori unit as (see Supporting Information for details):


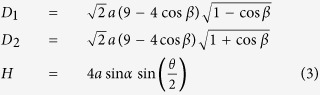


Note that *D*_1_ and *D*_2_ are diagonals of a diamond (i.e., the closed-loop element) and therefore always perpendicular to each other. In order to quantify the folding process, we define a non-dimensional parameter called ‘folding ratio’ as, 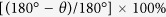, which varies from 0% (i.e., *θ* = 180°) to 100% (i.e., *θ* = 0°). In other words, 0% and 100% folding ratios correspond to two fully-folded configurations of the proposed construction.

The cross-sectional area of the closed-loop element, *S*, defined as the area of the polygon formed by intersecting the closed-loop element with a plane normal to its height, is constant through the height of the closed-loop element. The volume of the closed-loop element, *V*, is the volume bounded by the constituting first-order elements. Figure 4(b) depicts the variation of the cross-sectional area and volume of the closed-loop element (respectively normalized by *a*^2^ and *a*^3^) as functions of the folding ratio, respectively, presented for four different values of *α* ranging from 30° to 75°. The results are plotted using the analytical expressions of area and volume derived in the Supporting Information. As the folding ratio increases, the normalized area rises from zero (i.e., fully-folded configuration) up to a turning point, and then decreases due to the auxetic behavior of closed-loop element in both diagonal directions (will be discussed later). This is then followed by a plateau regime as the closed-loop element reaches the other fully-folded configuration. The critical folding ratio associated with the turning point decreases significantly for higher values of *α*. Similar behavior is observed for the variations of the normalized volume, except the fact that at 100% folding ratio, the volume becomes zero due to the fully-folded configuration of the closed-loop element.

Next, for an uniaxial out-of-plane load, we calculate the Poisson’s ratio of the closed-loop element in *D*_1_ and *D*_2_ directions (since they are always perpendicular to each other), defined as 
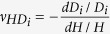
, where *i* = 1 or 2. Differentiating Equation (3) with respect to the folding angles and plugging the results into the above equations yield the following closed-form expressions for Poisson’s ratios:


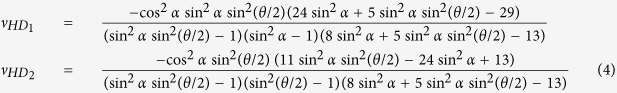


It is noteworthy that although these formulations were derived for a single closed-loop unit, they still hold true for the infinite periodic metamaterial. This is due to the fact that the calculations were performed on an RVE, which can be tessellated in diagonal (i.e., D_1_ and D_2_) and out-of-plane directions [as the “lattice vectors”]^30^ to form the final configuration of the metamaterial.

Figure 4(c) shows the dependence of Poisson’s ratio on the folding ratio in two orthogonal in-plane directions (i.e., *D*_1_ and *D*_2_), for four different values of *α* ranging from 30° to 75°. 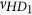 is negative for the entire range of folding ratio and *α*, with a significantly pronounced auxetic response at greater values of *α*. In contrast, 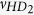 has a positive infinity value at 0% folding ratio [theoretically, the denominator of 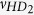 becomes zero at 0% folding ratio, see Equation (4)], which then reduces to 0 at 100% folding ratio. For 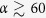, this involves exhibiting a negative Poisson’s ratio after a certain folding ratio. Insets in Fig. 4(b,c) illustarete the effect of changing *α* in the geometry and folding procedure of unit-cell. Figure 4(d) shows folding of a sample closed-loop element demonstrated under loading in out-of-plane compression and in-plane stretching along the direction of *D*_1_ (see Supporting Information for details). For this sample, *α* = 60° and the fully-folded states are achieved at *β* = 180° − 2*α* = 60° (or *θ* = 0°) and *β* = 180° (*θ* = 180°), as shown under out-of-plane compression and in-plane stretching experiments, respectively. Note that the closed-loop element, shown in Fig. 4(d) tessellates the 3D space regardless of folding level – see Supplementary Fig. S3.

Next, we investigated the force required to attain a desired level of folding for each building block of the cellular metamaterial under two loading directions (i.e., out-of-plane and in-plane). We assumed that each building block is made of rigid plates, connected together at straight creases modeled as linear torsional springs^15^ with spring constant per unit crease length of *k*(N). Also, as mentioned earlier, we idealized a building block of the cellular metamaterial as an infinite array of closed-loop elements stacked on top of each other, and analyzed the RVE. In the Supporting Information, we derived the following analytical expressions for the folding force on the RVE under out-of-plane and in-plane loadings using the principle of minimum total potential energy:


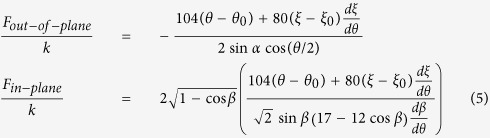


where *F*_*out*–*of*–*plane*_ and *F*_*in*–*plane*_ denote the folding forces for out-of-plane and in-plane loading directions, respectively, *θ*_0_ and *ξ*_0_ are the free angles of horizontal and inclined torsional springs, respectively (i.e., the angles at which no potential energy is stored in the springs), and *dξ*/*dθ* and *dβ*/*dθ* can be calculated using Equation (1).

Figure 5(a) shows the plots of normalized out-of-plane and in-plane folding forces, versus the folding ratio for different values of *α*, while the free angle of the torsional springs is kept constant as *θ*_0_ = 90° (i.e., 50% folding ratio; *ξ*_0_ can be calculated from Equation (1) by plugging *θ*_0_ instead of *θ*). In addition, for *α* = 60°, we plotted the normalized out-of-plane and in-plane folding forces versus the folding ratio for a set of *θ*_0_ varying between the extreme cases, *θ*_0_ = 0° and *θ*_0_ = 180°, Fig. 5(b). The results show a so-called “bistable“ behavior for 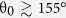 in out-of-plane loading, and for 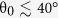 under in-plane loading. For example, the sample with θ_0_ = 170° exhibits local extremum points at 20% (local maximum) and 66% (local minimum) folding ratios when subjected to out-of-plane loading. This reveals the two stable configurations – one at the initial state (i.e., F/k = 0) where the folding ratio is 5.5%, and – the other one at the local minimum point at 66% folding ratio. We should note that the structure will go to the “local minimum” point (i.e., 66% folding ratio) only if the load is still there (i.e., a pre-load), otherwise, if we remove the load, the structure will always go back to its stable state at zero force (i.e., 5.5% folding ratio) after going through a “snap-through”^29^. This bistability in the response highlights the potential of the proposed cellular metamaterials for energy absorption, energy harvesting, and impact mitigation applications^31^,^32^,^33^. Next, we compare out-of-plane and in-plane loading responses for an RVE with *α* = 60° and *θ*_0_ = 90°, see Fig. 5(c). These calculations show that except for folding ratios greater than 78%, the in-plane force associated for achieving a specific folding ratio is lower than the out-of-plane force for the same value of folding ratio. This means that for folding ratios smaller than 78%, it is easier to fold the structure under in-plane loading (compared to an out-of-plane loading), while the opposite is true for folding ratios greater than 78%. Additionally, the inset of the figure shows that the folding ratio corresponding to the point at which the two curves meet [shown by a hollow circle in Fig. 5(c)], decreases with increasing *α*, making the out-of-plane force smaller than the in-plane force for a wider span of the folding ratio.

In summary, in this paper we propose an Origami-based paradigm of constructing cellular materials which are capable of undergoing large reversible deformation while exhibiting highly nonlinear auxeticity, bistability and topological locking. Particularly, the locking phenomena is used as a platform for scaling up these structures in a systematic modular fashion into larger cellular structures with single force activation without taking recourse to any special structural or surface modifications. The self-locking is achieved using an applied force on the structure. In the Supporting Information we discussed the force required for achieving the initial self-locking under different loading types and geometrical parameters. Thus, in summary, this present work sets forth an important avenue of novel cellular metamaterial design based on both self-similar and self-locking assembly.

## Methods

### Fabrication of the Origami-based elements and structures

All the elements and structures were fabricated out of paper (thickness ~ 0.01 in), where the cuts and crease lines were made using a Silhouette CAMEO cutting machine (Silhouette America, Inc., Lindon, UT).

### Tensile tests

We first subjected the prototype under out-of-plane compression using an Instron 5582 testing machine with a 1 kN load cell. Next, we manually applied in-plane tension using a force-gauge to directly measure the tensile force. The experiments were videotaped in order to qualitatively compare the results between the unlocked and locked states of the structure (see Supporting Information video).

## Additional Information

**How to cite this article:** Kamrava, S. *et al*. Origami-based cellular metamaterial with auxetic, bistable, and self-locking properties. *Sci. Rep.* **7**, 46046; doi: 10.1038/srep46046 (2017).

**Publisher's note:** Springer Nature remains neutral with regard to jurisdictional claims in published maps and institutional affiliations.

## Supplementary Material

Supplementary Information

Supplementary Video

## Figures and Tables

**Figure 1:**
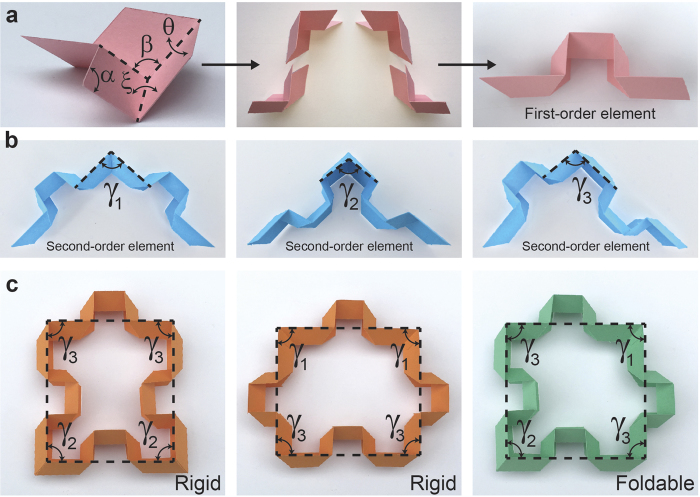
(**a**) (left image) The Miura-ori can be described by constant angle of *α* and the single degree of freedom (DOF) which can be defined in terms of dihedral angles, *θ*, and *ξ*, and the angle between mountain and front valley folding lines, *β*. (middle image) Two Miura-ori units are first positioned in a zigzag pattern, then mirrored to form a symmetric structure. (right image) ‘First-order element’, used in developing the Origami-based cellular metamaterial. (**b**) First-order elements are attached together in three different ways to make a ‘second-order element’ with internal angles, *γ*_1_, *γ*_2_, and *γ*_3_. (**c**) From all possible closed-loop elements, formed by using second-order elements, only one arrangement leads to a rigid-foldable geometry while the other are all rigid.

**Figure 2:**
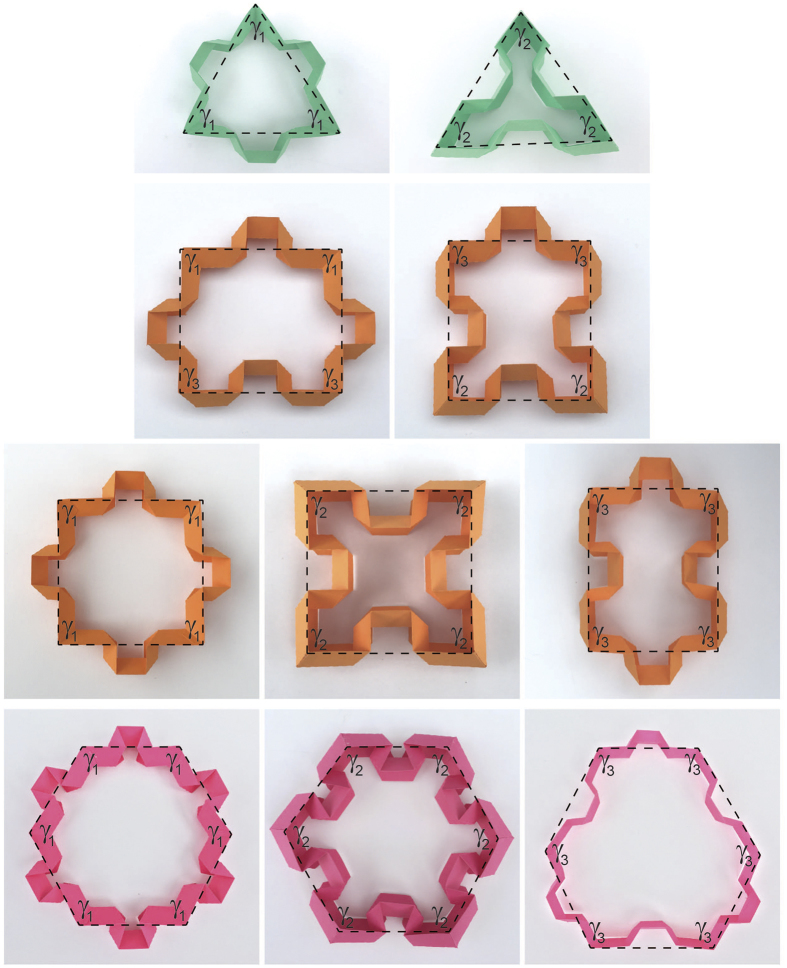
All possible configurations of triangular, quadrilateral, and hexagonal closed-loop elements (the only 2D shapes which can individually tessellate the 2D space to form periodic geometries), formed by different types of second-order elements.

**Figure 3:**
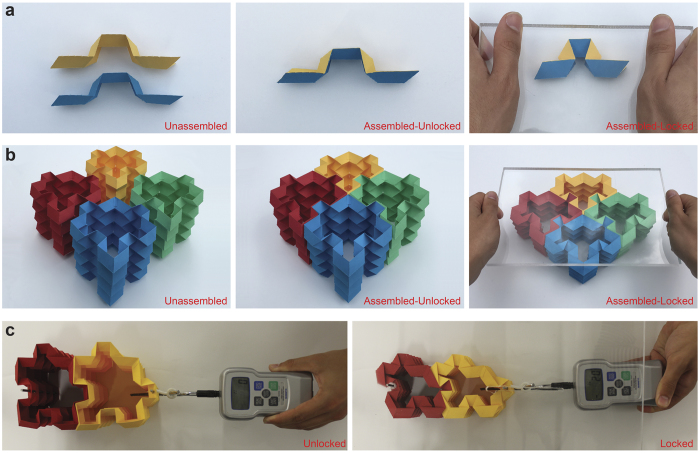
(**a**) Assembly and locking procedure for two first-order elements. (**b**) The assembly and self-locking feature of the first-order elements are transferred to the building blocks. This forms the final assembly of the Origami-based cellular metamaterial. (**c**) Measuring the resisting force for unlocked and locked states of two building blocks of the Origami-based cellular metamaterial, where the unlocked configuration exhibits no resisting force while in the locked state the structure shows noticeable resisting force before locking fails.

**Figure 4:**
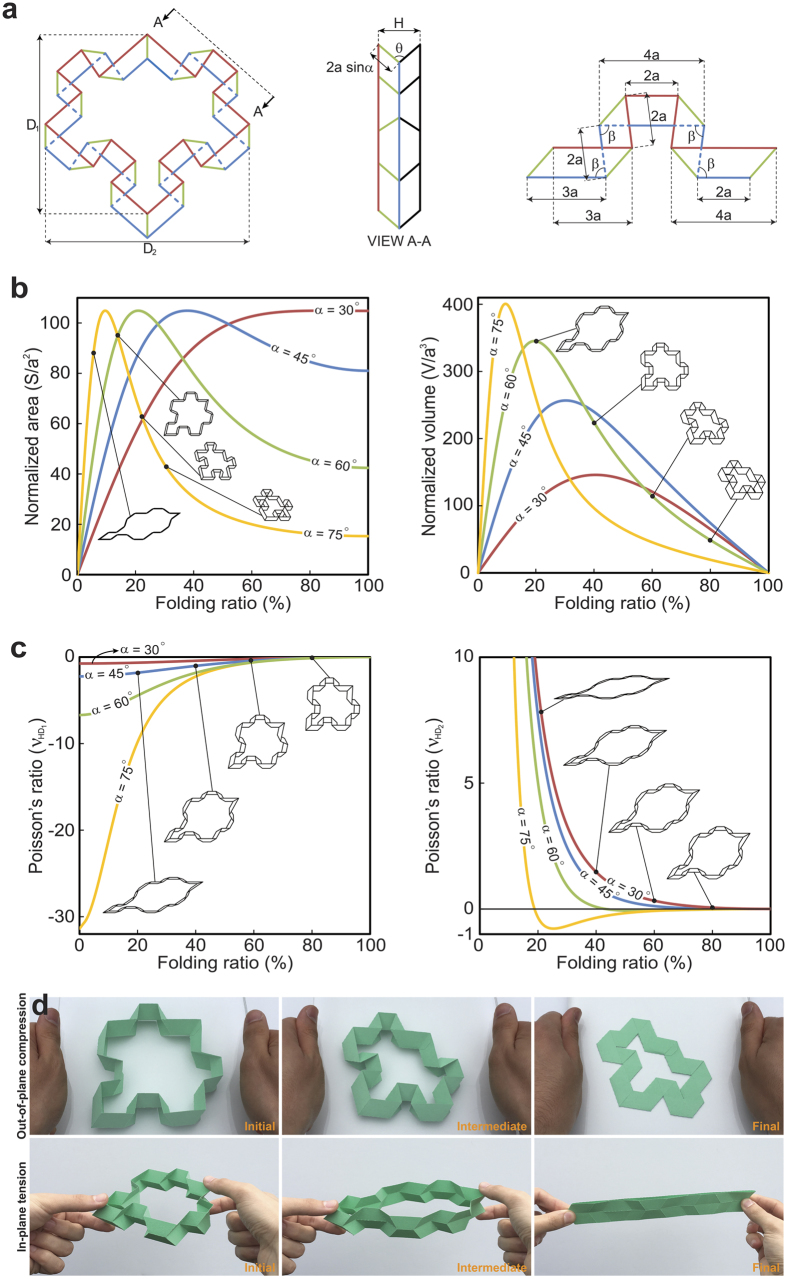
(**a**) Front and side views of the closed-loop element, as well as geometrical characteristics of the first-order element. The structural organization of the first-order element (as well as the closed-loop element) can be defined by two constant values related to the topology of the underlying Miura-ori unit, length a and angle *α*, and one variable angle which can be chosen between *β, θ*, and *ξ* representing the structure’s single degree of freedom. (**b**) Variations of cross-sectional area and volume of the closed-loop element (respectively normalized by *a*^2^ and *a*^3^) with respect to the folding ratio. (**c**) Plots of Poisson’s ratio versus folding ratio for in-plane diagonal directions, *D*_1_ and *D*_2_, while the insets in (**b**,**c**) show the folded configurations for *α* = 75°, 60°. 45°, 30° at the specified points. (**d**) Rigid-foldability of the closed-loop element under out-of-plane and in-plane loadings (i.e., two orthogonal directions).

**Figure 5:**
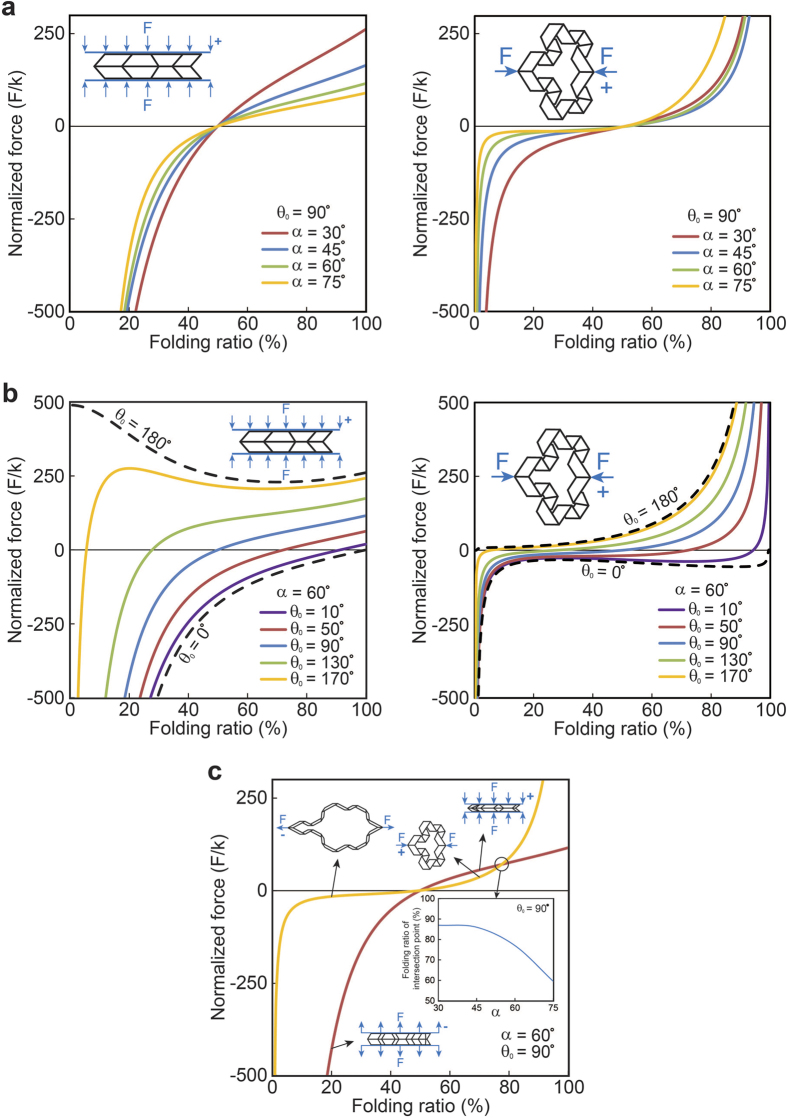
(**a**) The normalized out-of-plane and in-plane folding forces (i.e., F/k, where F is applying force and k is torsional spring constant per unit crease length) versus the folding ratio for different values of the angle, *α*, ranging from 30° to 75°, while the torsional springs are assumed to be free at 50% folding ratio [or equally *θ*_0_ = 90°, and *ξ*_0_ can be calculated from Equation (1)]. (**b**) The normalized out-of-plane and in-plane folding forces versus the folding ratio for a constant value of *α* = 60°, with *θ*_0_ varying between the extreme cases, *θ*_0_ = 0° and *θ*_0_ = 180°. (**c**) Comparison between out-of-plane and in-plane folding forces for an RVE with *α* = 60° and *θ*_0_ = 90°. The sub-plot presents the folding ratio versus *α*, for the point at which the out-of-plane and in-plane forces are equal.
