# Supplementary material for: Origami-based cellular metamaterial with auxetic, bistable, and self-locking properties
Source: Sci Rep. 2017 Apr 7;7:46046. doi: 10.1038/srep46046 (PMC5384242; doi:10.1038/srep46046)
Supplement: Supplementary Information [file srep46046-s1.pdf]

Supplementary Information

Origami-based cellular metamaterial with auxetic, bistable, and self-locking properties

Soroush Kamrava[1], Davood Mousanezhad[1], Hamid Ebrahimi[1], Ranajay Ghosh[2], and Ashkan Vaziri[1,*]

[1] Department of Mechanical and Industrial Engineering,
Northeastern University, Boston, MA 02115, USA

[2] Department of Mechanical and Aerospace Engineering,

University of Central Florida, Orlando, FL 32816, USA

[*] vaziri@coe.neu.edu

1. Geometrical characteristics

1.1. Miura-ori unit: angle $\boldsymbol{\beta}$

The configuration of a Miura-ori unit at an arbitrary level of folding can be fully quantified by an angular value defining the coordinate of the single degree-of-freedom (DOF) of the unit. This angular value can be chosen between the two dihedral angles, $\theta \in [0^o, 180^o]$, and $\xi \in [0^o, 180^o]$, or the angle between the mountain and valley folds, $\beta \in [180^o - 2\alpha, 180^o]$, see **Figure S1(a)**. As mentioned in the manuscript, due to the geometrical organization of the unit and rigidity assumption, only one of these angles is independent which can then be used as the coordinate of the single DOF of the unit for our analysis. To this end, considering θ as an independent parameter, we can obtain β and ξ as functions of θ (i.e., β and ξ will be considered as dependent parameters). We now begin the analysis by calculating the vectors $\overrightarrow{AB}$ and $\overrightarrow{AC}$ as the following [see Figure S1(a)]:

$$\overrightarrow{AB} = +L\cos\phi\,\vec{\imath} + L\sin\phi\,\vec{\jmath}$$
$$\overrightarrow{AC} = -L\cos\gamma\,\vec{\imath} - L\sin\gamma\,\vec{k} \quad (1)$$

where L is the edge length of the four identical parallelograms forming the Miura-ori unit, and $\vec{\imath}$, $\vec{\jmath}$, and $\vec{k}$ are unit vectors along the x, y, and z directions, respectively. Now, the following expression defines the angle between the vectors $\overrightarrow{AB}$ and $\overrightarrow{AC}$:

$$cos^{-1}\left(\frac{\overrightarrow{AB}.\overrightarrow{AC}}{|\overrightarrow{AB}||\overrightarrow{AC}|}\right) = 180^o - \alpha \quad (2)$$

Next, substituting supplementary Equation 1 into supplementary Equation 2 will result in the following relation between angles ϕ, γ, and α:

$$\cos\phi\ \cos\gamma = \cos\alpha \quad (3)$$

Now, considering the isosceles triangles, *ABF* and *AED* [see Figure S1(b)], the following relations can be obtained for the angles θ and ϕ:

$$\sin(\theta/2) = \frac{\overline{DE}/2}{\overline{AE}}$$
$$\sin\phi = \frac{\overline{BF}/2}{\overline{AB}} \quad (4)$$

where $\overline{DE}$ is the length of the edge DE (similarly for other edges). We should note that $\overline{AE} = \overline{AB}\sin\alpha$, and $\overline{BF} = \overline{DE}$, which by substituting into supplementary Equation 4 will result in the following:

$$\sin\alpha\ \sin(\theta/2) = \sin\phi \quad (5)$$

Finally, Figure S1(a) shows that $\beta = 180^o - 2\gamma$, which by using supplementary Equations 3 and 5 will result in the following equation for β:

$$\beta = 180^o - 2cos^{-1}\left(\frac{cos\alpha}{\sqrt{1 - sin^2\alpha\, sin^2(\theta/2)}}\right) \tag{6}$$

1.2. Miura-ori unit: angle ξ

In order to obtain a closed-form expression for the angle, ξ, as a function of θ, we first translate (with no rotations) the coordinate system of Figure S1(a) from point *A* to point *M*, see Figure S1(c). Note that the angle ξ is basically the angle between vectors $\overrightarrow{GA}$ and $\overrightarrow{GH}$. We now begin the analysis by obtaining the coordinates of points *G*, *A*, and *H* with respect to the new coordinate system located at point *M*, as the following:

$$G = \begin{bmatrix} X_G \\ Y_G \\ Z_G \end{bmatrix} = \begin{bmatrix} L\cos\alpha\cos\phi \\ L\cos\alpha\sin\phi \\ 0 \end{bmatrix}$$

$$A = \begin{bmatrix} X_A \\ Y_A \\ Z_A \end{bmatrix} = \begin{bmatrix} L\sin(\beta/2) \\ 0 \\ L\cos(\beta/2) \end{bmatrix} \tag{7}$$

$$H = \begin{bmatrix} X_H \\ Y_H \\ Z_H \end{bmatrix} = \begin{bmatrix} -L\sin(\beta/2) + 2L\cos\alpha\cos\phi \\ 2L\cos\alpha\sin\phi \\ L\cos(\beta/2) \end{bmatrix}$$

Note that we employed the relation, $\overline{NH} = 2\overline{MG} = 2L\cos\alpha$, to derive the set of coordinates presented in supplementary Equation 7. Next, using supplementary Equation 7 the vectors, $\overrightarrow{GA}$ and $\overrightarrow{GH}$, will be determined as the following:

$$\overrightarrow{GA} = \begin{bmatrix} L\sin(\beta/2) - L\cos\alpha\cos\phi \\ -L\cos\alpha\sin\phi \\ L\cos(\beta/2) \end{bmatrix}$$

$$\overrightarrow{GH} = \begin{bmatrix} -L\sin(\beta/2) + L\cos\alpha\cos\phi \\ L\cos\alpha\sin\phi \\ L\cos(\beta/2) \end{bmatrix} \tag{8}$$

Next, we calculate the angle ξ as:

$$\cos\xi = \left(\frac{\overrightarrow{GA}\,.\,\overrightarrow{GH}}{|\overrightarrow{GA}||\overrightarrow{GH}|}\right) = \frac{\cos\beta - \cos^2\alpha + 2\cos\alpha\cos\phi\sin(\beta/2)}{1 + \cos^2\alpha - 2\cos\alpha\cos\phi\sin(\beta/2)} \tag{9}$$

which can further be simplified (by using supplementary Equations 5 and 6) into the following:

$$\xi = \cos^{-1}\left(\frac{1 - (1 + \cos^2\alpha)\ \sin^2(\theta/2)}{1 - \sin^2\alpha\ \sin^2(\theta/2)}\right) \tag{10}$$

1.3. Closed-loop elements foldability vs. rigidity

As we mentioned in the manuscript, the only possible closed-loop element (i.e., polygon) with rigid-foldability property, formed by different types of connection introduced in Figure 1(b), is the one highlighted in green in Figure 1(c) – right image. For instance, here, we will prove that the quadrilateral element shown in Figure S2 (a), is completely rigid, though it does not violate the geometrical constraint on internal angles, presented by Equation (2) of the manuscript.

We begin the analysis by noting that the length of the edges of this closed-loop element, $\overline{AB}$, $\overline{CD}$, $\overline{AD}$, and $\overline{BC}$, can be obtained as the following [see Figure S2(a), and **Figure 4(a)**]:

$$\begin{aligned}\overline{AB} &= \overline{CD} = 8a - 4a\,cos\,\beta \\ \overline{AD} &= \overline{BC} = 9a - 4a\,cos\,\beta\end{aligned} \tag{11}$$

where α and β are defined in Figure 4(a). Furthermore, the following relation must hold for the edges and internal angles of the quadrangle, $ABCD$:

$$\overline{AB} = \overline{CD} - \overline{AD}\cos\beta - \overline{BC}\cos\beta = 8a - 22a\cos\beta + 8a\cos^2\beta \tag{12}$$

Next, $\overline{AB}$ in supplementary Equation 11 and supplementary Equation 12 must be equal, which results in the following:

$$8a - 4a\, cos\, \beta = 8a - 22a \cos \beta + 8a \cos^2 \beta \tag{13}$$

which holds true only for $\beta = 90^o$. Therefore, supplementary Equation 13 is not valid for all values of β (equally, every level of folding). In other words, the element is not foldable (equally, it is rigid). Similarly, we can show that all the elements in Figure 2 are rigid, however, they can be used as building blocks to construct rigid tessellations such as the well-known 'Kagome' structure made from triangular and hexagonal elements, which is shown in Figure S2 (b).

1.4. Closed-Loop element cross-sectional area and volume

Cross-sectional area of closed-loop element is equal to the area of the underlying diamond formed by the surrounding dashed lines shown in Figure 1(c) – right image. Thus, the area can be calculated as the following (see Figure 4(a)):

$$cross - sectional\ area = 0.5\, D_1 D_2 = a^2 (9 - 4 \cos \beta)^2 \sin \beta \tag{14}$$

where D_1 and D_2 ar diagonals of the diamond [see Figure 4(a)]. Furthermore, the volume of the element can be calculated as the following:

$$volume = (cross - sectional\ area)\ .\ H = 4a^3 (9 - 4 \cos \beta)^2 \sin \beta \sin \alpha \sin(\theta/2) \tag{15}$$

Finally, the expressions derived for cross-sectional area and volume of the closed-loop element are normalized with respect to a^2 and a^3, respectively.

1.5. Periodic unit cells

The foldable unit cell remains periodic in different folding levels. **Figure S3** shows the tessellation of four unit cells, while they are still periodic, in folding ratios 100%, 35%, 14%, and 9%.

2. Force-folding relations

Here, we calculate both out-of-plane and in-plane folding forces required to attain a desired level of folding. We first assume that the structure is made of rigid plates, held together at the crease lines which are modeled as linear torsional springs with spring constant per unit length of k (N). We then idealize our tubular structure as an infinite array of closed-loop elements stacked on top of each other. This allows us to perform our analytical calculations at a single unit of repetition which can serve as the classical representative volume element (RVE). The RVE in our current analysis is a single polygonal closed-loop element with crease lines at top and bottom (i.e., at the lines joining to the upper and lower elements) modelled as torsional springs with spring constant per unit length of $k/2$ (N). Now, we calculate the total potential energy stored in torsional springs, and the external work done by external forces, while the DOF of the RVE goes from θ_0 (i.e., initial folding angle of torsional springs) to θ (i.e., desired level of folding):

$$U_{first-order-element} = 2 \times \frac{1}{2} k(\theta - \theta_0)^2 \times (3a + 2a + 4a + 2a + 2a) + \frac{1}{2} k(\xi - \xi_0)^2 \times (16a)$$

$$U_{connecting-edges} = \frac{1}{2} k(\xi - \xi_0)^2 \times (8a) + \frac{1}{2} k\big(\pi - \xi - (\pi - \xi_0)\big)^2 \times (8a)$$

$$U_{total} = 4U_{first-order-element} + U_{connecting-edges} = 52\, ka(\theta - \theta_0)^2 + 40ka(\xi - \xi_0)^2 \tag{16}$$

and,

$$W_{out-of-plane} = F_{out-of-plane} \times \Delta H$$
$$= F_{out-of-plane} \times \big(4a \sin\alpha \sin(\theta_0/2) - 4a \sin\alpha\ \sin(\theta/2)\big)$$

$$W_{in-plane} = F_{in-plane} \times \Delta D_1$$
$$= F_{in-plane}$$
$$\times \left(\sqrt{2}\, a\, (9 - 4\cos\beta)\sqrt{1 - \cos\beta} - \sqrt{2}\, a\, (9 - 4\cos\beta_0)\sqrt{1 - \cos\beta_0}\right) \tag{17}$$

where $U_{first-order-element}$, and $U_{connecting-edges}$ are the total potential energy of the torsional springs located within each first-order element, and in the edges that connect the first-order elements together, respectively. Also, $W_{out-of-plane}$, and $W_{in-plane}$ are the total external work done by the applied external force, respectively during out-of-plane ($F_{out-of-plane}$), and in-plane ($F_{in-plane}$) loadings.

Finally, using the principle of minimum total potential energy (i.e., $\partial\pi/\partial\theta = 0$, where $\pi = U_{total} - W$), the normalized forces can be obtained as the following:

$$\frac{F_{out-of-plane}}{k} = -\frac{104(\theta - \theta_0) + 80(\xi - \xi_0)\dfrac{d\xi}{d\theta}}{2\sin\alpha\cos(\theta/2)}$$

$$\frac{F_{in-plane}}{k} = 2\sqrt{1-\cos\beta}\left(\frac{104(\theta - \theta_0) + 80(\xi - \xi_0)\dfrac{d\xi}{d\theta}}{\sqrt{2}\sin\beta\,(17 - 12\cos\beta)\dfrac{d\beta}{d\theta}}\right) \tag{18}$$

On studying the self-locking behavior of closed-loop element (and tubular elements), here we study the critical force at which self-locking first occur in the structure. To this end, we first define the self-locking critical force as the force (in-plane or out-of-plane) required to initiate self-locking behavior (i.e., $\beta = 90°$) in the structure. First of all, supplementary Equation 6 requires that for self-locking behavior to happen in the structure, the angle, α, must be greater than 45° (this can be obtained by substituting $\beta = 90°$ in supplementary Equation 6). Figure S4 shows the contour plots of the self-locking critical force as a function of θ_0 and α for out-of-plane (left) and in-plane (right) loadings. Note that for loadings greater than the critical force, the structure will go into the locked configuration; otherwise it will remain unlocked.

3. Experiments

We first fabricated the prototypes of closed-loop and tubular elements using papers (thickness ~0.01in). All cuts and perforations were made using a Silhouette CAMEO cutting machine (Silhouette America, Inc., Lindon, UT).

In order to demonstrate the self-locking behavior of the Origami-based cellular metamaterial we proposed in this paper, two tubular units (each unit is made of four closed-loop elements, stacked on top of each other) were used to perform uniaxial tensile tests for unlocked and locked configurations. For the case of locked configuration, we subjected the prototype under out-of-plane compression using an Instron 5582 testing machine with a 1kN load cell, while for the case of unlocked configuration, no loading was imposed to the structure in the vertical direction. Then, the structure was fixed at one end, while the other end was pulled uniaxially using a digital force gauge (which was implemented to measure the required in-plane force to detach the tubular elements), **Figure S5**. The results indicate that the required in-plane force to detach the elements is almost 0 N in the case of unlocked configuration, while it is around 35 N for the case of locked configuration (at an arbitrary level of folding). This remarkable difference in detaching force demonstrates the importance of self-locking feature of our proposed Origami-based cellular metamaterial. See the Supplementary Movie titled “Self-locking feature of the proposed Origami-based cellular metamaterial”.

Supplementary Movie legend

Self-locking is achieved by applying an out-of-plane compression which keeps the Origami-based cellular metamaterial at certain folding ratios in which the self-locking behavior is guaranteed. In this Movie, we compare unlocked and locked configurations of the Origami-based cellular metamaterial in term of resistance to separating in-plane force.

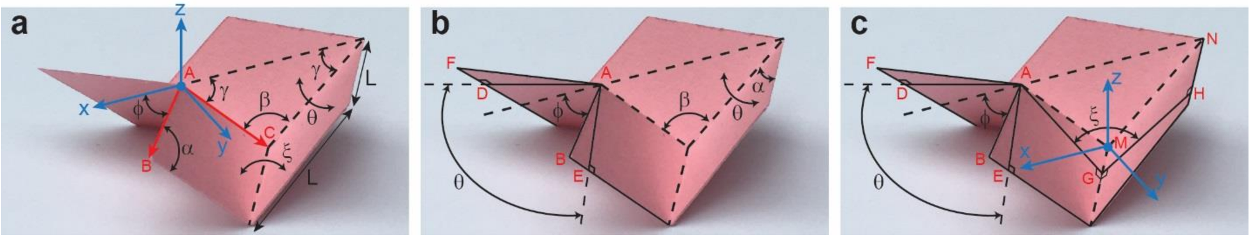

Figure S1. Geometrical characteristics of a Mira-ori fold at an arbitrary level of folding.

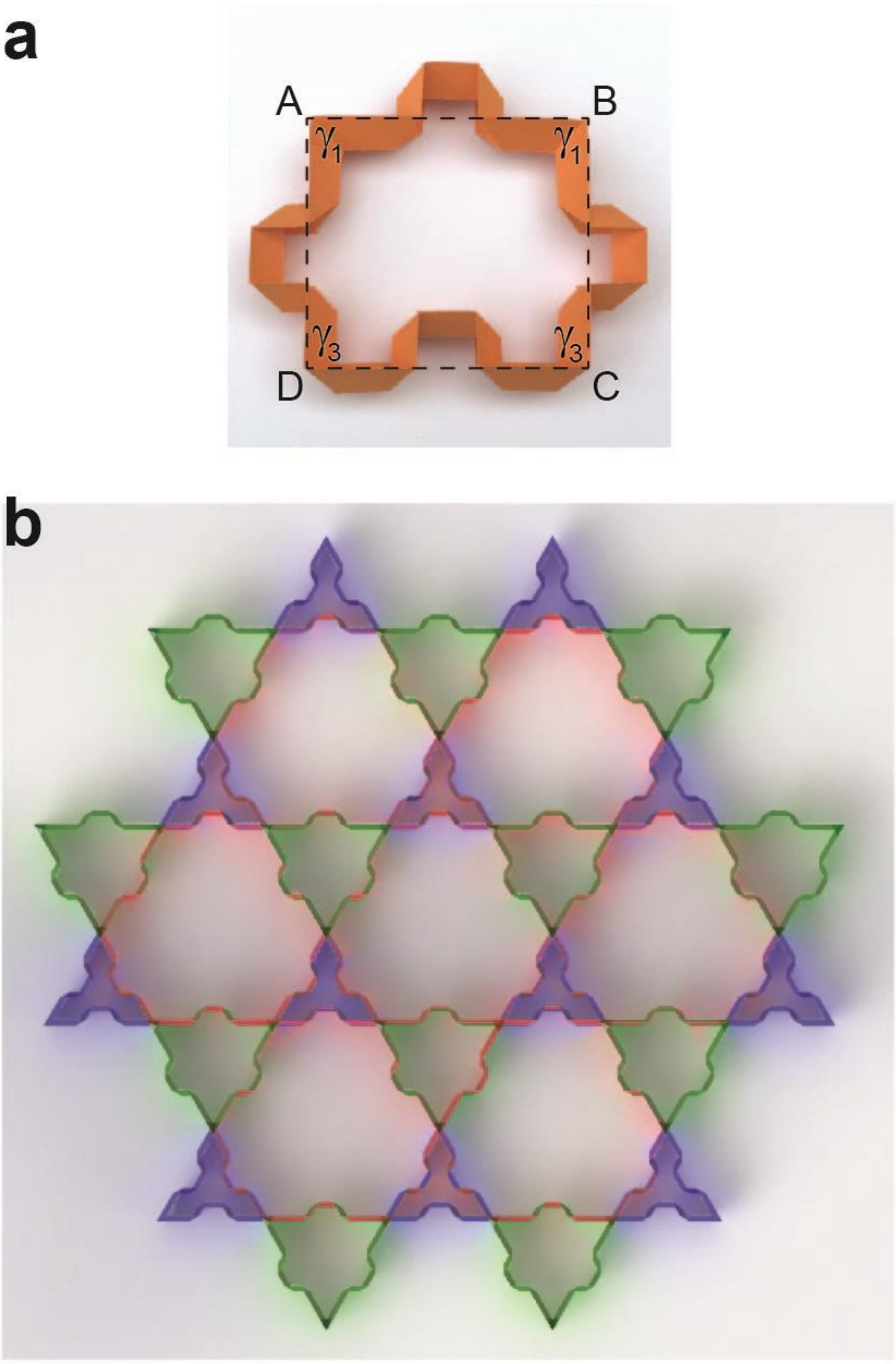

Figure S2. (a) Rigid quadrilateral closed loop element **(b)** ‘Kagome’ structure made from rigid triangular and hexagonal elements.

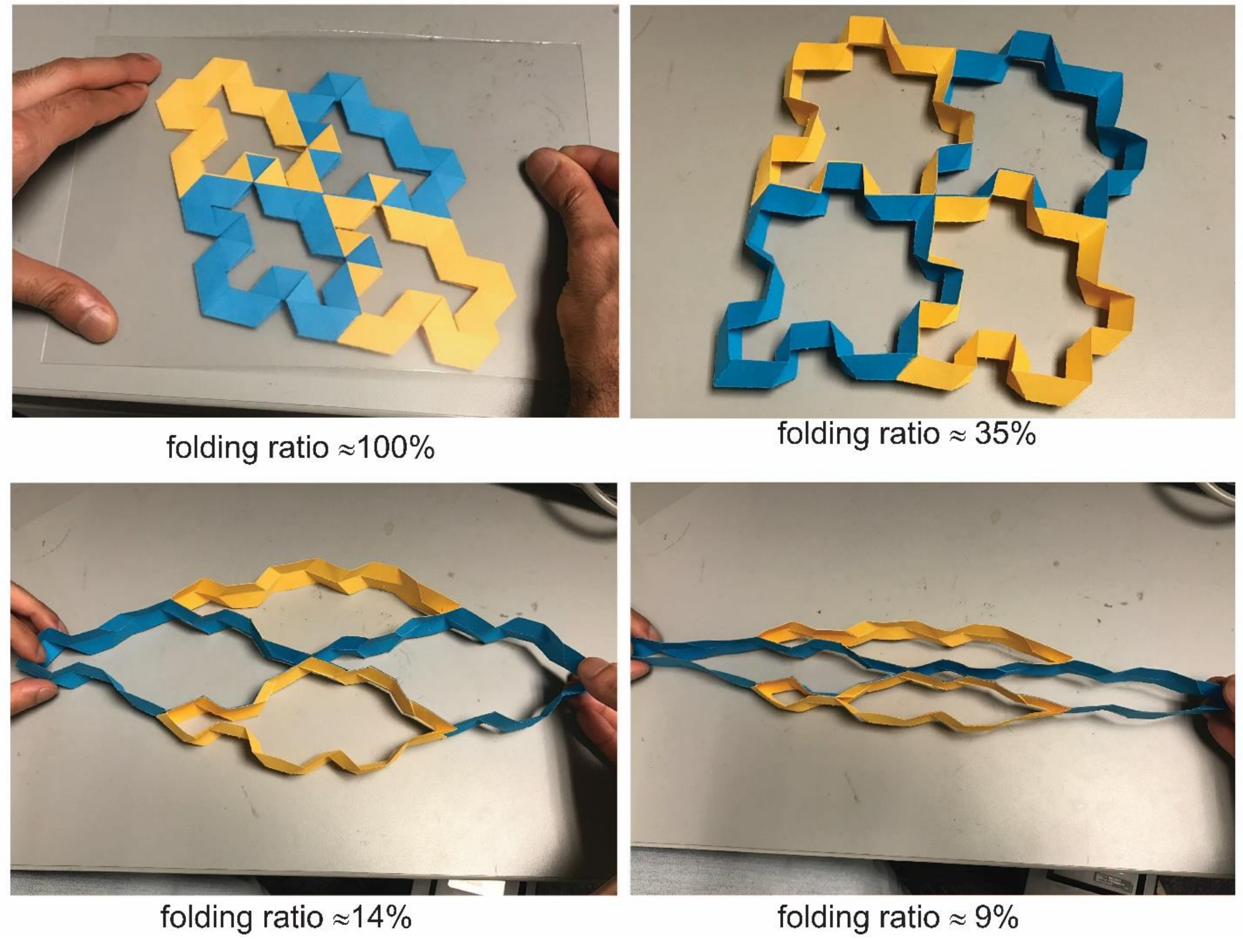

Figure S3. Periodic unit cells in folding ratios 100%, 35%, 14%, and 9%.

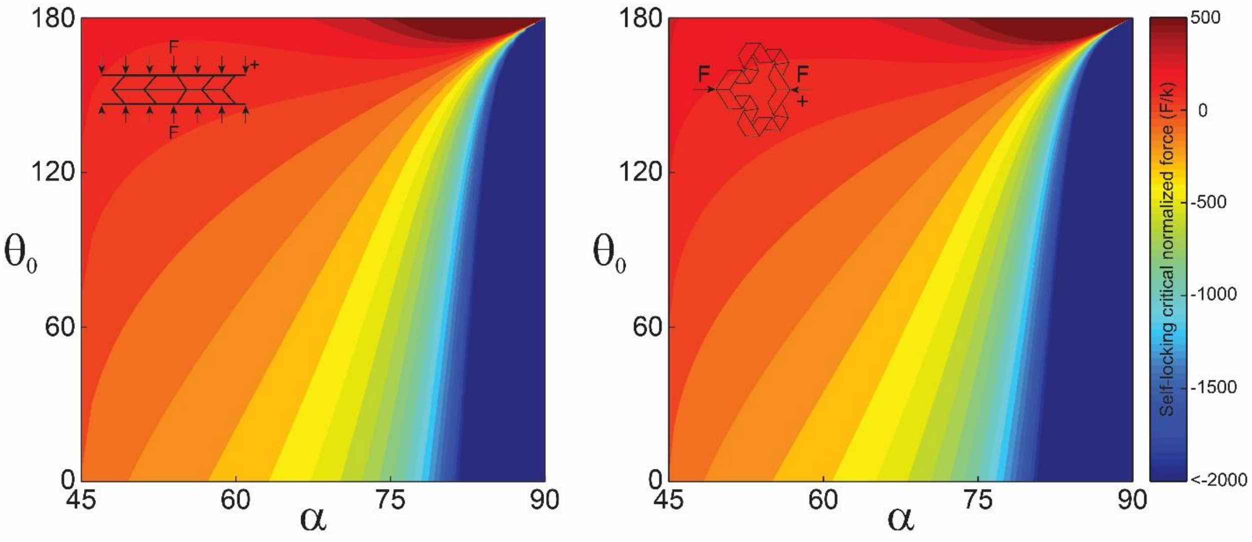

Figure S4. Self-locking critical normalized force (to achieve the locked configuration) as a function of θ_0 and α for out-of-plane (left) and in-plane (right) loadings.

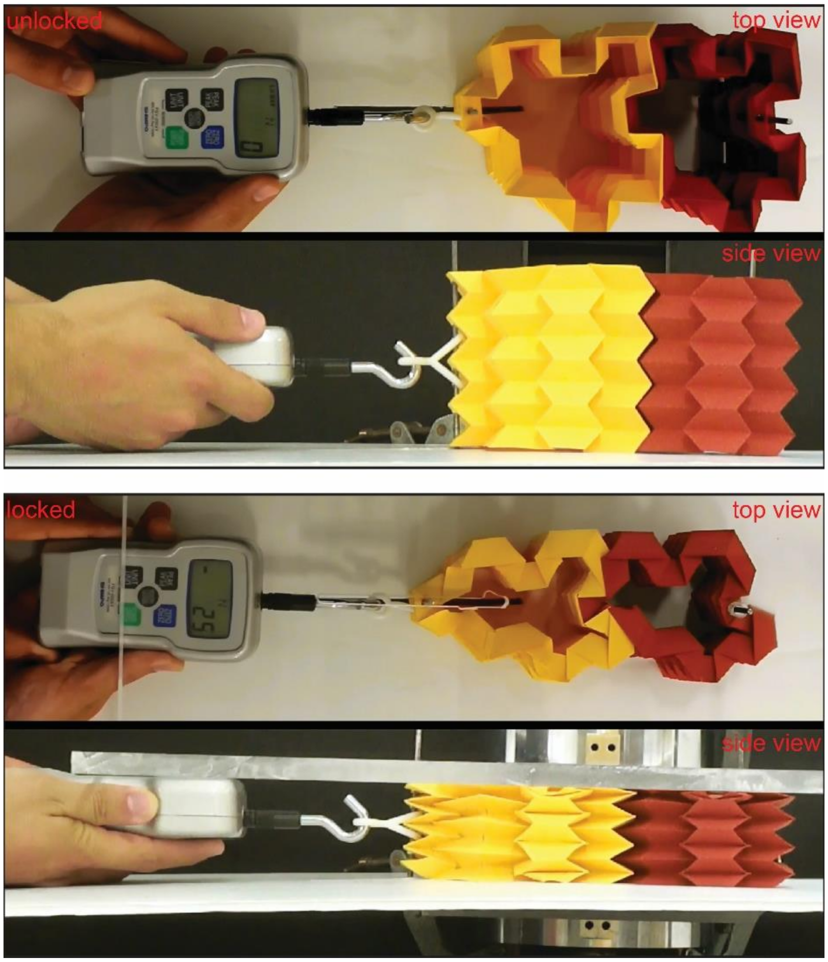

Figure S5. Demonstration of self-locking feature of our Origami-base cellular metamaterial through experiments.
